# Supplementary material for: Kinetics of Water-Induced Amorphous Phase Separation in Amorphous Solid Dispersions via Raman Mapping
Source: Pharmaceutics. 2023 May 2;15(5):1395. doi: 10.3390/pharmaceutics15051395 (PMC10223424; doi:10.3390/pharmaceutics15051395)
Supplement: Supplementary file 1 [file pharmaceutics-15-01395-s001.zip › Supporting Information.pdf]

pharmaceutics

Supplementary Information

Kinetics of Water-Induced Amorphous Phase Separation in Amorphous Solid Dispersions via Raman Mapping

Adrian Krummnow [1,2], Andreas Danzer [1], Kristin Voges [2], Samuel O. Kyeremateng [2,*], Matthias Degenhardt [2] and Gabriele Sadowski [1,*]

[1] TU Dortmund University, Department of Biochemical and Chemical Engineering, Laboratory of Thermodynamics, Emil-Figge-Str. 70, D-44227 Dortmund, Germany
[2] AbbVie Deutschland GmbH & Co. KG, Global Pharmaceutical R&D, Knollstraße, D-67061 Ludwigshafen am Rhein, Germany
* Correspondence: gabriele.sadowski@tu-dortmund.de (G.S.); Tel.: +49-231-755-2635; samuel.kyeremateng@abbvie.com (S.O.K.); Tel.: +49-621-589-4940

Keywords: phase behavior, miscibility gap, amorphous phase separation, kinetics, Raman imaging, confocal Raman spectroscopy, copovidone

Citation: Krummnow, A.; Danzer, A.; Voges, K.; Kyeremateng, S.O.; Degenhardt, M.; Sadowski, G. Kinetics of Water-Induced Amorphous Phase Separation in Amorphous Solid Dispersions via Raman Mapping. *Pharmaceutics* 2023, *15*, x. https://doi.org/10.3390/xxxxx

Academic Editors: María Teresa Viciosa, Madalena Dionísio and Hermínio P. Diogo

Received: 27 March 2023
Revised: 27 April 2023
Accepted: 28 April 2023
Published: 2 May 2023

Copyright: © 2023 by the authors. Submitted for possible open access publication under the terms and conditions of the Creative Commons Attribution (CC BY) license (https://creativecommons.org/licenses/by/4.0/).

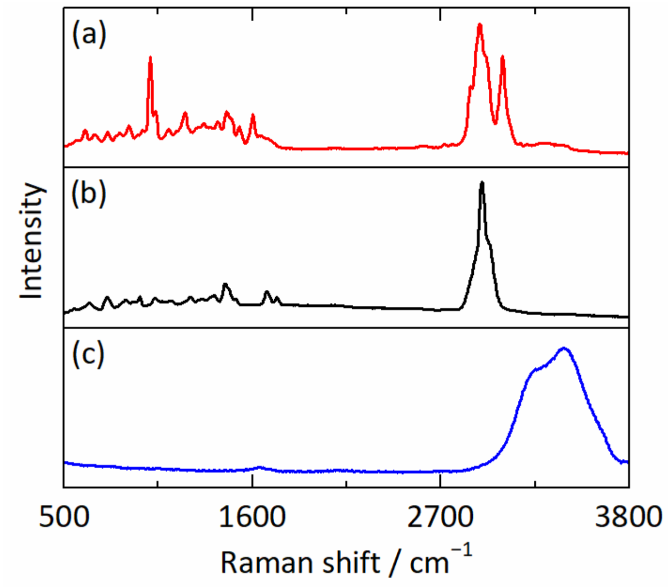

Figure S1. Pure component spectra of (**a**) amorphous RIT, (**b**) PVPVA, and (**c**) water at 25 °C.

Table S1. Compositions of samples for calibration spectra at 25 °C

	$w_{\mathbf{RIT}}$	$w_{\mathbf{PVPVA}}$	$w_{\mathbf{water}}$
Pure components	1	0	0
	0	1	0
	0	0	1
RIT/PVPVA	0.1	0.9	0
	0.2	0.8	0
	0.7	0.3	0
PVPVA/water	0	0.05	0.95
	0	0.10	0.90
	0	0.69*	0.31*
	0	0.85†	0.15†

* According to a gravimetric water sorption measurement after storage at 94% RH [11].
† According to a gravimetric water sorption measurement after storage at 74% RH in this work.

S1. Statistical Indicators

The statistical indicators R_i^2 (coefficient of determination), $RMSEC_i$ (Root Mean Square Error of Calibration), $RMSECV_i$ (Root Mean Square Error of Cross-Validation), $BiasC_i$ (Mean of the differences of calibration) and $BiasCV_i$ (Mean of the differences of Cross-Validation) were calculated for every component by using Equations S1-S5.

$$R_i^2 = 1 - \sum_k^{NP_i} \frac{w_{i,k}^{\text{meas}} - w_{i,k}^{\text{true}}}{w_{i,k}^{\text{true}} - \bar{w}_i^{\text{true}}} \quad \text{(S1)}$$

$$RMSEC_i = \sqrt{\frac{1}{NP_i} \sum_k^{NP_i} \left(w_{i,k}^{\text{meas}} - w_{i,k}^{\text{true}}\right)^2} \quad \text{(S2)}$$

$$RMSECV_i = \sqrt{\frac{1}{NP_i} \sum_k^{NP_i} \left(w_{i,k}^{\text{meas,CV}} - w_{i,k}^{\text{true}}\right)^2} \quad \text{(S3)}$$

$$BiasC_i = \frac{1}{NP_i} \sum_k^{NP_i} w_{i,k}^{\text{meas}} - w_{i,k}^{\text{true}} \quad \text{(S4)}$$

$$BiasCV_i = \frac{1}{NP_i} \sum_k^{NP_i} w_{i,k}^{\text{meas,CV}} - w_{i,k}^{\text{true}} \quad \text{(S5)}$$

$w_{i,k}^{\text{meas}}$ is the measured mass fraction, $w_{i,k}^{\text{meas,CV}}$ is the cross-validated measured mass fraction and $w_{i,k}^{\text{true}}$ is the true mass fraction at data point k of in total NP_i data points of component i. $\bar{w}_i^{\text{true}}$ is the mean true mass fraction of in total NP_i data points of component i. The statistical indicators for the calibration results are reported in Table S2.

Table S2. Calibration results in statistical indicators

	**RIT**	**PVPVA**	**water**
R_i^2	0.9994	0.9992	0.9996
$RMSEC_i$	0.0082	0.0112	0.0080
$RMSECV_i$	0.0118	0.0180	0.0137
$BiasC_i$	−0.0026	0.0064	−0.0038
$BiasCV_i$	−0.0010	0.0023	−0.0013

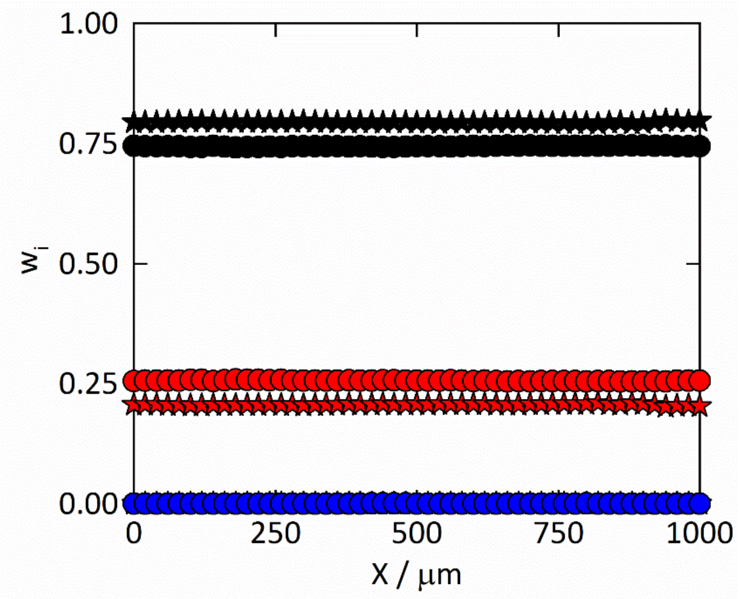

Figure S2. Mass fractions of RIT (red), PVPVA (black) and water (blue) in a freshly-prepared 20 wt% (stars) and 25 wt% (circles) DL RIT/PVPVA ASD obtained from 1000 µm line scans at 25 °C.

Author Contributions: Conceptualization, A.K., A.D., K.V., S.O.K., M.D. and G.S.; methodology, A.K., A.D., K.V., S.O.K., M.D. and G.S.; software, A.K. and A.D.; validation, A.K., A.D., K.V., S.O.K., M.D. and G.S.; formal analysis, A.K.; investigation, A.K.; resources, M.D. and G.S.; data curation, A.K. and A.D.; writing—original draft preparation, A.K.; writing—review and editing, A.K., A.D., K.V., S.O.K., M.D. and G.S.; visualization, A.K.; supervision, A.D., K.V., S.O.K., M.D. and G.S.; project administration, A.D., K.V., S.O.K., M.D. and G.S.; funding acquisition, M.D. and G.S. All authors have read and agreed to the published version of the manuscript.

Funding: This study was funded by AbbVie.

Institutional Review Board Statement: Not applicable.

Informed Consent Statement: Not applicable.

Data Availability Statement: Data is contained within the Article or Supplementary Material.

Acknowledgments: The authors gratefully thank Stefanie Dohrn for fruitful discussions related to this work.

Conflicts of Interest: This study was funded by AbbVie. AbbVie participated in the study design, research, data collection, analysis, and interpretation of data, as well as writing, reviewing, and approving the publication. A.K., K.V., S.O.K., and M.D. are AbbVie employees and may own AbbVie stock/options. G.S. is a professor, A.D. is a postdoc, and A.K. is a Ph.D. student at the Department of Biochemical and Chemical Engineering at TU Dortmund University, and they have no conflict of interest to report.

Disclaimer/Publisher's Note: The statements, opinions and data contained in all publications are solely those of the individual author(s) and contributor(s) and not of MDPI and/or the editor(s). MDPI and/or the editor(s) disclaim responsibility for any injury to people or property resulting from any ideas, methods, instructions or products referred to in the content.
